# Lead Transfer during Breastfeeding: A Start toward Filling in the Data Gaps

**DOI:** 10.1289/ehp.122-A26

**Published:** 2014-01-01

**Authors:** Julia R. Barrett

**Affiliations:** Julia R. Barrett, MS, ELS, a Madison, WI–based science writer and editor, has written for *EHP* since 1996. She is a member of the National Association of Science Writers and the Board of Editors in the Life Sciences.

Adverse health effects of lead have been detected at ever decreasing blood concentrations.^1^ Yet there is still a degree of mystery around the toxicokinetics of lead in the body—that is, the processes controlling a chemical’s uptake, biotransformation, distribution, and elimination, which ultimately determine how toxic it is.^2^ A new study in *EHP* provides insight into lead transfer from mothers to their children via breast milk.^3^

Lead is stored primarily in bone. Women’s blood lead levels rise during pregnancy and lactation due to increased bone turnover, which releases previously stored lead. Human milk is a proven route of lead exposure for breastfeeding infants, although the lack of information about lead toxicokinetics has made it impossible to clearly define any risk. Lead exposure *in utero* and during breastfeeding can cause adverse effects in children independent of any later exposure, so prevention is critical.^4^

Clinicians are generally advised that women with blood lead levels below 40 µg/dL can breastfeed without clinical harm to their children.^5^ The rationale is that only a tiny fraction of the lead in blood is bioavailable (i.e., biologically active).^6^ Human milk is low in protein, the molecular carrier of lead.^3^ Lead is therefore not expected to transfer heavily from blood into milk, and the milk-to-plasma (M/P) ratio for humans has been reported at less than 1.0.^5^ (A ratio of 1.0 would indicate equal concentrations of lead in milk and plasma.)

**Figure:**
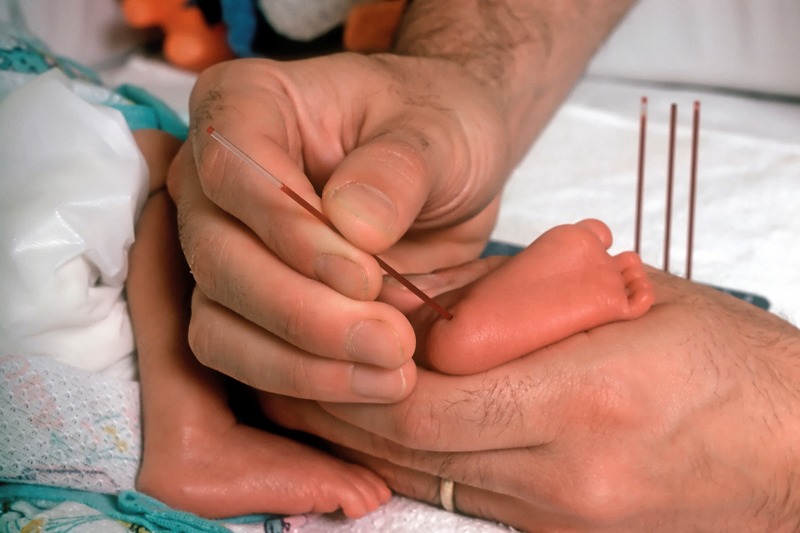
Given that human milk can be an important source of lead for nursing infants, early testing may be prudent for babies of highly exposed women. © RGB Ventures LLC dba SuperStock/Alamy

But the authors of the current study could find no clear justification for that number in humans. To validate the ratio, they used samples that had been collected in Mexico City in the late 1990s during a longitudinal study of lead biomarkers and reproduction. In that study 463 pregnant women provided blood and plasma samples during pregnancy and the first year postpartum. Breastfeeding mothers also provided milk samples 1 month postpartum. The current study focused on blood, plasma, and milk samples from 81 women; for a subsample of 60 mother–infant pairs, 3-month infant blood samples were available.

Lead concentrations were measured in all samples and were found to vary widely: 1.7–28.7 µg/dL, 0.03–0.5 µg/L, and 0.04–3.2 µg/L for maternal blood, plasma, and milk, respectively, and 0.5–14.5 µg/dL for infant blood. M/P ratios ranged from 0.6 to 39.8, averaging 7.7, with 97% of the ratios exceeding 1.0.

According to Claudia Gundacker, an associate professor at the Institute of Medical Genetics, Medical University Vienna, who was not involved with the study, further work is needed to substantiate the findings on the higher M/P ratio and determine what they might mean for advising pregnant and nursing women. “In general,” she says, “there is a gap of knowledge on lead cellular toxicokinetics and on transport of lead across membranes.” She says the major strength of the study is that breast milk lead was analyzed in relation to maternal plasma lead and whole blood lead, “which provides valuable information on the distribution of lead in the various compartments.”

Lead author Adrienne Ettinger, an assistant professor at the Yale Center for Perinatal, Pediatric and Environmental Epidemiology, stresses that the findings should not discourage women from breastfeeding. “However, if a woman is known to have lead exposure, … the infant’s blood lead levels should be checked earlier than they normally would be,” she says. That way, steps can be taken to reduce or eliminate exposure.

Children typically aren’t checked until they’re 9–12 months old. “In certain places,” Ettinger says, “they’re not being checked at all because they are considered ‘low risk’ [for lead exposure].”

Gundacker notes that the study raises many questions regarding the factors contributing to the huge variability in the observed M/P ratios, including genetic variation. Although the cross-sectional design of the study limits the conclusions that can be drawn, the variability of lead transfer from mothers to their children via breast milk may provide an impetus to reexamine current advice, she says.
